# Effect of Whey Protein Purity on the Characteristics of Algae Oil-Loaded Encapsulates Obtained by Electrospraying Assisted by Pressurized Gas

**DOI:** 10.3390/nano12183096

**Published:** 2022-09-07

**Authors:** Cristina Prieto, Emma Talón, Caciano Zapata Noreña, Jose M. Lagaron

**Affiliations:** 1 Novel Materials and Nanotechnology Group, Institute of Agrochemistry and Food Technology (IATA), Spanish Council for Scientific Research (CSIC), Calle Catedrático Agustín Escardino Benlloch 7, 46980 Paterna, Spain; 2 Bioinicia R & D Department, Bioinicia S.L., Calle Algepser No. 65, Nave 3, Polígono Industrial Táctica, 46980 Paterna, Spain; 3 Institute of Food Science and Technology, Federal University of Rio Grande do Sul, Av. Bento Golçalves, No. 9500, Porto Alegre CEP 91501-970, Brazil

**Keywords:** whey protein, PUFAs, algae oil, nanoencapsulation, functional food, effect of wall material

## Abstract

In this paper, the effect of protein purity in three different whey protein grades on the characteristics of algae oil encapsulates obtained via room-temperature electrospraying assisted by pressurized gas (EAPG) encapsulation process was studied. Three different commercial grades of whey protein purity were evaluated, namely 35, 80, and 90 wt.%. Oil nanodroplets with an average size of 600 nm were homogeneously entrapped into whey protein microparticles 3 µm in size. However, the sphericity and the surface smoothness of the microparticles increased by increasing the protein purity in the grades of whey protein studied. The porosity of the microparticles was also dependent on protein purity as determined by nitrogen adsorption–desorption isotherms, being smaller for larger contents of protein. Interestingly, the lowest extractable oil was obtained with WP35, probably due to the high content of lactose. The peroxide values confirmed the superior protective effect of the protein, obtaining the smallest peroxide value for WP90, a result that is consistent with its reduced porosity and with its lower permeability to oxygen, as confirmed by the fluorescence decay–oxygen consumption method. The accelerated stability assay against oxidation confirmed the higher protection of the WP80 and WP90. In addition, the increased content in protein implied a higher thermal stability according to the thermogravimetric analysis. These results further confirm the importance of the adequate selection of the composition of wall materials together with the encapsulation method.

## 1. Introduction

Encapsulation processes can be described as the technology used to entrap a bioactive compound into a wall material [1] in order to protect it from different environmental factors such as temperature, oxygen, humidity, and pH, among others, which may provoke a strong reduction in bioactivity and bioavailability [2]. The selection of the coating material is a key phase in the design of an encapsulation system [3]. The wall material can improve the core material stability, mask undesirable aromas and flavors, provide controlled release and increase bioavailability [4,5]. The encapsulating matrices available for food applications are limited to edible, preferably inexpensive, biocompatible, and biodegradable materials. Additionally, the encapsulation matrix should have a bland flavor, high solubility, emulsification properties, and film-forming and drying characteristics [6]. Wall materials approved for food use include natural gums, carbohydrates, lipids, and some proteins [7]. Among proteins, milk proteins, including caseins, whey proteins, and milk fat globule membrane proteins, present high solubility and low viscosity and may be selected as wall materials for hydrophobic compounds, and thanks to their amphiphilic character, they are excellent interfacial agents, being able to be used in the formation and stabilization of emulsions. Moreover, milk proteins can establish covalent or electrostatic complexes with bioactive compounds, or entrap them via the formation of gels. In addition, milk proteins have good film-forming and mechanical properties, high gas barrier properties, and high resistance to organic solvents and oils or fats [8,9].

Whey protein is a blend of globular proteins that constitutes 20% of the proteins in milk, and is commercially obtained as a byproduct of cheese manufacturing [10]. Whey protein composition varies depending on milk composition and the method of casein removal, but normally has 50% of the solids, which consist of fat, lactose, proteins, minerals, and vitamins. Two types of whey protein are commercially available, i.e., whey protein concentrate and whey protein isolate, which contain approximately between 35% and 80% and over 90% of protein, respectively [11]. These compounds have GRAS status, low cost, and great nutritional value, and are common emulsifying, gelling, and thickening ingredients in foods. Additionally, they have previously been shown to have antioxidant activities and barrier effects [12]. However, variations in the composition of whey proteins can affect the quality of the encapsulates [13].

Recent studies have proven the potential of whey protein as a wall material for the protection of long-chain polyunsaturated omega-3 fatty acids (PUFAs) via the innovative high-throughput electrospraying assisted by pressurized gas (EAPG) [14,15]. This encapsulation process is based on the nebulization of an encapsulant and bioactive solution by a pneumatic injector using compressed gas that atomizes this, and the resulting nebulized droplets are further exposed to an electric field, which further splits, favors encapsulation, and dries the droplets. During the EAPG process, the solvent is evaporated at room temperature and the encapsulated material is then recovered as a free-flowing powder [14]. The use of room temperature ensures the protection of the bioactive compounds, whereas the use of voltage results in high encapsulation efficiency and controlled particle size distribution [16].

Despite the compositional effects on physicochemical and functional properties of whey proteins being extensively described in the literature for encapsulates produced by spray drying [6,17,18], no information regarding the composition effect on particles produced via electrospraying or room-temperature EAPG has been found. The fact that these encapsulation processes are carried out at room temperature may affect the formation of the crust and the porosity of the wall material, and together with the compositional effect of the whey protein could affect the oxidative stability of the oil inside the particle. For this reason, the objective of this research was to evaluate, for the first time, the influence of protein purity in whey protein composition on the characteristics of the encapsulates produced by room-temperature EAPG encapsulation technology, using algae oil enriched in DHA as a highly sensitive bioactive compound. For this study, three different compositions of whey protein were considered, i.e., 35, 80, and 90 wt.%. Characterization of the obtained particles was performed in terms of their morphology, porosity, oil entrapment, and oxidative and thermal stability.

## 2. Materials and Methods

### 2.1. Materials

Commercial whey proteins with different levels of purity were supplied by Beurrespa (Madrid, Spain). Table 1 shows the composition of the grades of whey protein used according to the product data provided by the supplier. Despite the fact that proportion between components changes across the three grades, the main differences were found to lay in the percentage of lactose, with the concentration of the rest of the components being below 10%. Therefore, it is anticipated that the main differences in performance will be ascribed to the content of protein and lactose in the encapsulates.

DHA-enriched algae oil was provided by Q’omer Bioactive Ingredients (Valencia, Spain). As claimed by the supplier, the algae oil has a DHA content of 40 wt.%. The oil was kept under vacuum in the dark at −20 °C. Hydrochloric acid 37 vol.% and Span 20 were from Sigma Aldrich (St. Louis, MO, USA). Barium chloride dihydrate (reagent grade), iron (III) chloride hexahydrate (PRS), chloroform (99%), and methanol (reagent grade) were provided by Panreac Química SLU (Barcelona, Spain). Iron (II) sulfate heptahydrate (analytical grade) was purchased from Labkem-Labbox (Mataró, Spain). Ammonium thiocyanate (99%) and isopropanol (99.5%) were obtained from Acros Organics (Geel, Belgium). 2,2,4-trimethylpentane (≥99.0%) was obtained from Honeywell (Morristown, NJ, USA). Ethanol 96 vol.% was supplied by Laboratorios e Industrias Noriega S.L. (Oviedo, Spain). Deionized water was employed throughout this investigation.

### 2.2. Preparation of the Emulsion

The same emulsion formulation was used to encapsulate the algae oil in the different grades of whey protein. The aqueous phase of the emulsion consisted of an aqueous solution of the different whey protein grades at a concentration of 22.5 wt.%. The dispersed phase was prepared by dissolving the Span 20 at a concentration of 9.1 wt.% in the algae oil. The mass ratio between the dispersed phase and the continuous phase was 11:89, resulting in a biopolymer-to-algae-oil mass ratio of 2:1. The dispersed phase was gradually added to the aqueous solution with constant nitrogen bubbling. The emulsion was homogenized with an UltraTurrax T-25 (IKA, Staufen, Germany) at 17,000 rpm for 5 min, and 5 min of ultrasounds (90%) (Bandelin Sonopuls, Berlin, Germany) with constant nitrogen bubbling, with the emulsion being immersed in an ice bath to prevent temperature rising during the homogenization.

### 2.3. Emulsion Droplet Size

A Mastersizer 2000 (Malvern Instruments, Ltd., Worcestershire, UK) was used to measure the emulsion droplet size distribution. Recirculating water (3000 rpm) was used to dilute the emulsions, until the sample reached an obscuration of 12%. The refractive indices of sunflower oil (1.469) and water (1.330) were used for the particle and dispersant, respectively. Results were expressed as the average Sauter diameter (D3,2) of three measurements.

### 2.4. EAPG Process

The freshly prepared emulsion was instantly processed by EAPG using the proprietary Capsultek^TM^ pilot plant from Bioinicia S.L. (Valencia, Spain). This pilot unit consists of a nebulizer, the atomized droplets of which are subjected to an electric field; an evaporating chamber; and a cyclonic collector, as explained elsewhere [14,15,19,20]. The encapsulates were produced at monitored ambient conditions, i.e., 25 °C and 30% relative humidity (RH), maintaining the emulsion with constant nitrogen bubbling to minimize oil oxidation. The emulsion flowrate was 1 mL/min, and the injector worked with an assisted air pressure of 10 L/min and an electric voltage of 10 kV. The produced particles were collected as free-flowing powder every 20 min from the cyclone and stored in airtight flasks, at −20 °C in the dark until further analysis. In addition, whey protein solutions without oil were also processed as the control sample.

### 2.5. Morphology Characterization

An S-4800 FE-SEM (Hitachi High Technologies Corp., Tokyo, Japan) was used to analyze the particles’ morphology. Scanning electron microscopy (SEM) was performed with an electron beam acceleration of 5 kV, with the samples being sputtered with a gold/palladium layer. Particle diameters were measured using Image J Launcher v1.41 (National Institutes of Health, Bethesda, MD, USA). The data, expressed as average size and standard deviation, were based on measurements from at least 100 particles.

Transmission electron microscopy (TEM) in a JEM 1010 (JEOL Ltd., Tokyo, Japan) was used to study the internal morphology of the particles. Samples were included in LR white resin, and ultrathin sections after polymerization were cut using an ultramicrotome and deposited over the TEM grid [21].

### 2.6. Extractable Oil from the Particles

UV-Vis spectrophotometry was used to quantify the extractable oil (EO). For that purpose, 25 mg of encapsulates were washed with isooctane for 30 s and filtered. The amount of the algae oil in the filtrate was quantified at 285 nm in a UV4000 spectrophotometer (Dinko Instruments, Barcelona, Spain). A standard curve of algae oil in isooctane was built at concentrations between 0.1 and 0.5 mg/mL (y = 0.3068x, R^2^ = 0.99).

The percentage of extractable oil was calculated according to Equation (1) as the quotient of the amount of extractable oil detected in the filtrate (A) divided by the theoretical amount of algae oil present in the encapsulates (B). Analyses were performed in triplicate. It should be taken into account that the thorough extraction process with isooctane in such small particles may also potentially remove some oil from inside the particles.

EO = (A/B) · 100(1)

### 2.7. Oxidative Stability Tests under Ultraviolet Radiation

The accelerated oxidative stability test was performed under ultraviolet light (UV) for 10 days at ambient temperature and relative humidity. A total of 10 g of sample was located on Petri dishes 20 cm below the ultraviolet lamp. The lamp used for this assay was an Ultra-Vitalux (300 W) (OSRAM, Garching, Germany) which produces an intense blend of radiation very similar to that of natural sunlight [22,23]. Aliquots were taken on a daily basis for analysis via attenuated total reflectance Fourier transform infrared spectroscopy (ATR-FTIR) and the peroxide value (PV).

### 2.8. Peroxide Value Determination

The methodology to perform the peroxide value (PV) determination has been described elsewhere [14]. The oil was recovered from the particles using the Bligh and Dyer method [24], and the PV was estimated following the method described by Shantha and Decker [25]. Briefly, 0.4 g of BaCl_2_·2H_2_O was dissolved in 50 mL of distilled water. Separately, a ferrous solution was prepared by dissolving 0.5 g of FeSO_4_·7H_2_O in 50 mL of distilled water. The barium solution was slowly added to the ferrous solution under magnetic stirring, then 2 mL of HCl 10 N were added. The BaSO_4_ precipitate was filtered to obtain a clear FeCl_2_ solution, which was stored in an opaque flask. Freshly prepared FeCl_2_ solution was used in each procedure. To prepare the complexing agent, 30 g of NH_4_SCN were dissolved in 100 mL of distilled water.

To determine the peroxide value of the neat algae oil, 8 mg of algae oil were dissolved in 1 mL of ethanol 85%. In case of particles, the oil was extracted according to the Bligh and Dyer method [24]. For this, 0.5 g were dissolved in 1 mL of deionized water. A total of 0.5 mL of the previous solution was mixed with 1.5 mL of isooctane/isopropanol (2:1 *v*/*v*) mixed in the vortex and centrifuged at 1000 rpm for 4 min. The organic phase containing the oil was removed for further analysis.

After that, an aliquot of 200 µL of the oil solutions was mixed with 9.6 mL of chloroform-methanol (7:3 *v*/*v*). Then, 50 µL of NH_4_SCN was added and mixed in the vortex. After 5 min of reaction protected from light, the absorbance was measured at 500 nm against a blank containing all reagents, except the sample.

To construct the standard curve of absorbance versus Fe^3+^ concentration, a standard solution of iron (III) chloride was prepared. A total of 0.121 g of FeCl_3_·6H_2_O was dissolved in water and made up to 25 Ml. A total of 0.5 mL of the previous solution was made up to 50 mL with chloroform/methanol (7:3 *v*/*v*). Standard Fe^3+^ samples containing 0–40 µg Fe^3+^ were analyzed following the previous method by UV-Vis spectrophotometry at 500 nm, with the calibration curve being: y = 0.0158x − 0.0059, R^2^ = 0.998.

Equation (2) was used to calculate the peroxide value, which was expressed as milliequivalents of peroxides per kilogram of oil.

PV = [(As − Ab)/m] · V/(2 · 55.84 · m_0_ · S) (2)

where As and Ab are the absorbance of the sample and blank, respectively; m is the slope of the calibration curve; m_0_ is the weight sample of oil; 55.84 g/mol is the atomic weight of iron; S is the volume of the aliquot of the oil solution; V is the volume used to dissolve the oil. Analyses were performed in triplicate.

### 2.9. Attenuated Total Reflectance Fourier Transform Infrared (ATR-FTIR)

A Tensor 37 FT-IR Spectrometer (Bruker, Ettlingen, Germany) coupled with the ATR sampling accessory Golden Gate (Specac Ltd., Orpington, UK) was used to study the ATR-FTIR spectra of the samples. A total of 50 mg of sample was deposited on the diamond crystal for the analysis. The spectra were acquired in the range 4000–600 cm^−1^, by averaging 10 scans, with a 4 cm^−1^ resolution. Spectral data were analyzed using the OPUS 4.0 software (Bruker, Ettlingen, Germany). Origin 8.5 (OriginLab, Northampton, MA, USA) was used for peak deconvolution using the Bigaussian fitting function.

### 2.10. Headspace Oxygen Volume Depletion

A multichannel oxygen meter OXY-4 mini (PreSens Precision Sensing GmbH, Regensburg, Germany) was employed to determine the oxygen barrier capacity of the different grades of whey protein studied. The fluorescence method was used to measure the headspace oxygen volume depletion over 140 h at room temperature and 0% RH, following the methodology described elsewhere [14]. A total of 2.5 g of sample, or the equivalent amount for the neat oil, was deposited inside a 100 mL Schleck flask. Results were normalized to the initial oxygen volume and are the average of two measurements. The standard deviation among the measurements was lower than 2%.

### 2.11. Thermogravimetric Analysis (TGA)

A 550-TA Instruments thermogravimetric analyzer (New Castle, DE, USA) was used to evaluate the thermal stability of the samples. A total of 5 mg of sample was placed on a platinum pan and kept under 50 mL/min of air and heated between 25 and 700 °C, at a heating rate of 10 °C/min. Results analysis was performed using the Trios software (TA Instruments, New Castle, DE, USA).

### 2.12. Nitrogen Adsorption and Desorption Isotherms

A Tristar II 3020 device (Micromeritics Instrument Corporation, Norcross, GA, USA) was used to determine the nitrogen adsorption–desorption isotherms at the temperature of −196 °C (nitrogen boiling point). The specific surface areas and the average pore diameter of the samples were obtained using the Brunauer–Emmett–Teller (BET) method [26], whereas the pore size distribution curves were determined using BJH method [27] between 17 and 3000 Å.

## 3. Results and Discussion

The aim of this work was to compare the morphological characteristics and the oxidative and thermal stability of the algae oil encapsulated into three whey protein grades with different protein purities.

### 3.1. Morphology

**Figure 1:**
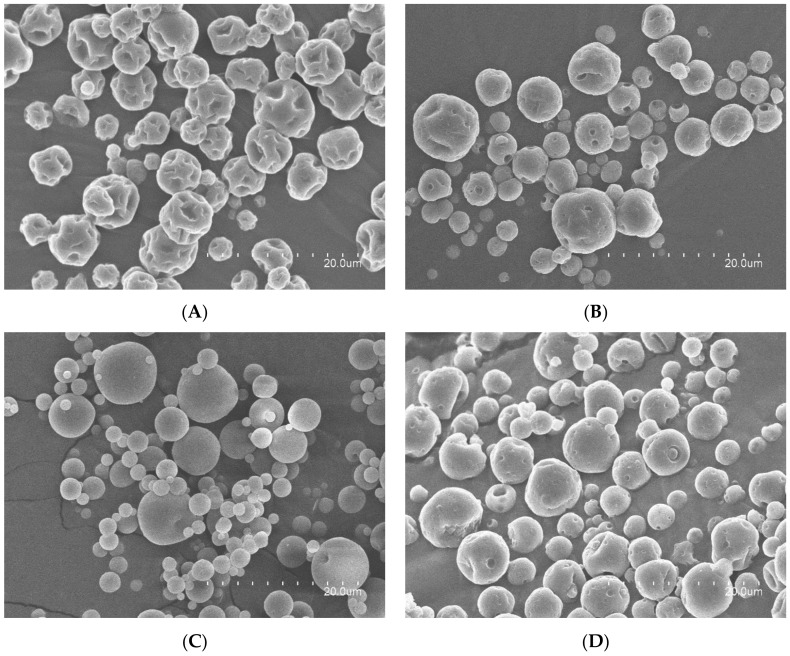

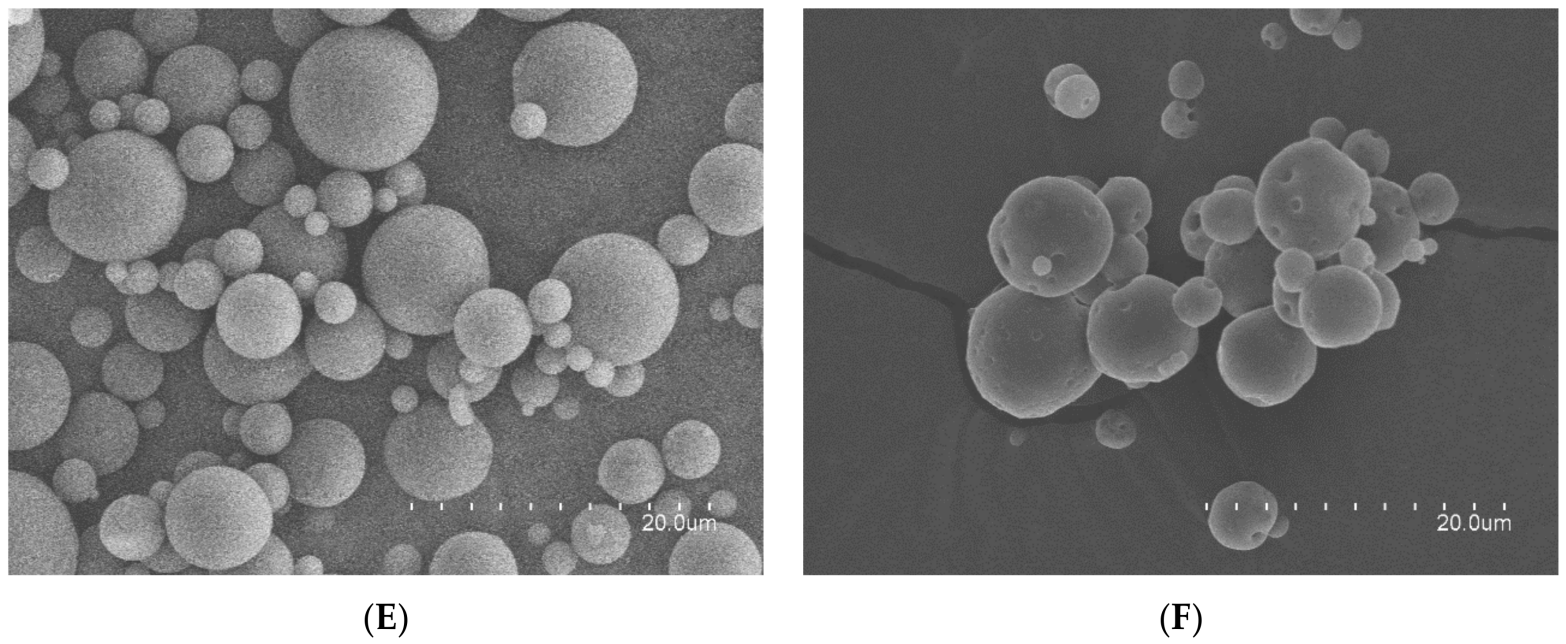
Scanning electron microscopy (SEM) micrographs: (**A**) neat WP35 particles; (**B**) WP35—algae oil (2:1) particles; (**C**) neat WP80 particles; (**D**) WP80—algae oil (2:1) particles; (**E**) neat WP90 particles; (**F**) WP90—algae oil (2:1) particles.

A comparison of the morphology of the particles obtained with the different whey proteins is shown in Figure 1. It is possible to observe the effect of the protein purity in the roughness and in the sphericity of the microparticles. Thus, particles were more spherical and less wrinkled as the protein purity increased, as shown in captions A, C, and E of Figure 1, for neat WP35, WP80, and WP90, respectively. Similar observations were made by other authors using different drying techniques. Both et al. reported a decrease in roughness by increasing protein content when producing microparticles with different whey protein:lactose ratios by spray drying [28]. Choi et al. also reported a decreased roughness by increasing protein content when producing whey protein microparticles by spray drying [17]. Perez-Masiá et al. obtained a similar morphology, i.e., particles with a reduced degree of roughness, when preparing microparticles of whey protein concentrate by electrospraying and nanospray drying [29]. Rodrigues et al. also obtained spherical and smooth particles when electrospraying whey protein isolate [30], similarly to the morphologies obtained by EAPG for the same grade of whey protein.

Regarding particle size, protein purity did not show a significant effect, with the average particle size being around 3.5 µm (neat WP80 2.16 ± 1.29 µm, neat WP90 3.72 ± 2.02 µm, neat WP35 4.44 ± 1.76 µm). Rosenberg et al. also did not observe an effect of protein purity on particle size when they processed different grades of whey protein by spray drying obtaining sizes between 1–25 µm in all cases [6]; however, they obtained larger sizes than by EAPG. Rodrigues et al. reported obtaining nanometric whey protein isolate particles when they electrosprayed a 18% whey protein isolate solution in ethanol [30]. This reduced diameter in comparison to EAPG could be due to the reduced solid content and also to the use of ethanol as solvent. Perez-Masiá et al. reported larger average diameters and broader size distribution with the spray-drying technique than with electrospraying when preparing whey protein concentrate microparticles by these two techniques [29].

The incorporation of the algae oil led to particles with decreased roughness in case of WP35, as can be observed in Figure 1B. However, in case of WP80 and WP90, the incorporation of the oil produced the generation of some dents. In these structures, the presence of the oil could provoke a loss of mechanical resistance. Choi et al. also reported the generation of some wrinkles on the microparticles’ surfaces when encapsulating conjugated linoleic acid into whey protein concentrate and whey protein isolate via spray drying [17]. The obtained particle size for the three types of microparticles encapsulating algae oil is reflected in Table 1. The incorporation of oil did not also generate a significant effect on particle size.

Herein-produced particles with WP80 encapsulating algae oil presented a rougher surface and a larger particle size in comparison to the one previously reported by Prieto et al. via EAPG [14], in which the whey protein concentrate grade used, also with 80 wt.% purity, was heat-stabilized by the manufacturer. The morphological differences between these two whey proteins could then arise from different physicochemical properties.

**Figure 2:**
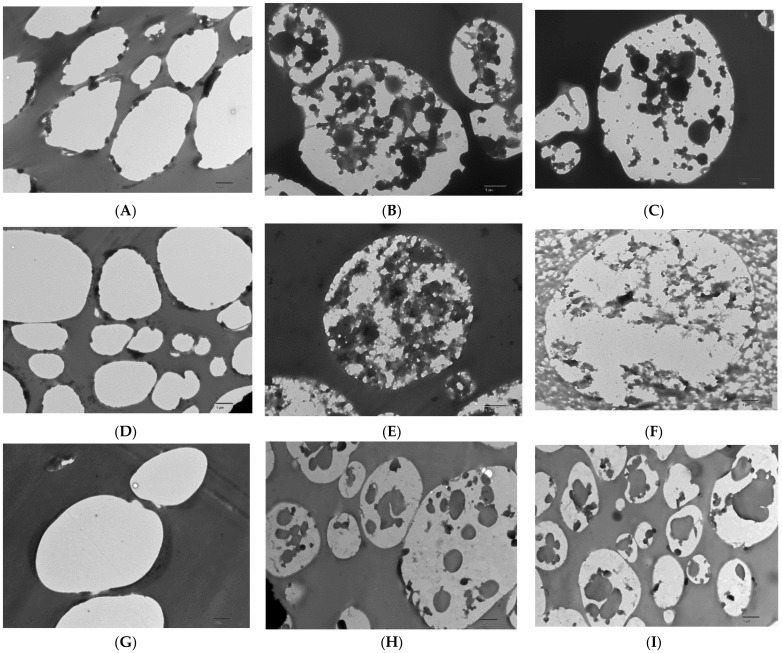
Transmission electron microscopy (TEM) micrographs: (**A**) neat WP35 particles; (**B**,**C**) WP35—algae oil (2:1) particles; (**D**) neat WP80 particles; (**E**,**F**) WP80—algae oil (2:1) particles; (**G**) neat WP90 particles; (**H**,**I**) WP90—algae oil (2:1) particles. Image scale is 1 µm.

A comparison between the internal morphology of the neat microparticles and the internal morphology of the microparticles encapsulating algae oil is shown in Figure 2. This characterization is relevant for the discussion about the algae oil retention and oxidative stability. TEM micrographs of the microtomed particles showed microparticles with similar size and morphology, as observed in SEM. However, no internal structure was observed for the neat microparticles, whereas spongy structures with oil pockets were observed for the microparticles encapsulating the oil. A good dispersion of the oil within the solid matrix is thought to maximize oil retention, protect against oxidation, and enhance bioavailability [18,31,32]. The size of these submicron cavities seems to be influenced by the protein purity, and by the droplet size of the emulsion obtained for each formulation, as shown in Table 2. Images demonstrate that the oil pockets presented sizes ranging from 194 nm to 1342 nm and with an average value of 600 nm, in concordance with the droplet size of the emulsion. The size of the submicron cavities was larger than the one reported by Prieto et al. when encapsulating algae oil into heat-stabilized 80 wt.% protein whey protein concentrate, maybe because of the better emulsification capacity of the heat-stabilized whey protein concentrate grade [14]. However, the cavity size was similar to the one obtained with an EPA oil and the same WP80 encapsulant [15]. Nevertheless, it was possible to observe an effect of the protein purity on the oil pockets’ distribution inside the particle, since the particles produced with the WP90 presented the oil cavities more concentrated in the center of the particle in comparison with WP80 and WP35, where the oil is more homogeneously distributed along the particle. This could indicate that it is possible to modify the internal structure of the particle, increasing the purity of the protein from a homogeneous distribution to a core shell structure. This phenomenon could be due to the effect of the voltage attracting the protein to the surface of the droplet during the EAPG process.

### 3.2. Extractable Oil

The amount of extractable oil was quantified by UV-Vis spectrophotometry using an extraction method with isooctane as organic solvent. The results are presented in Table 2. From this table, it was observed that the extractable oil was dependent on the composition of the whey protein, this being the lowest for the WP35 sample. This could be due to the larger content of lactose, which could act as filler [18,33]. Young et al. also reported enhanced encapsulation efficiencies by increasing the content of lactose in the whey protein, when encapsulating anhydrous milk fat via spray drying [6]. Gómez-Mascaraque et al. obtained similar oil-retention values when encapsulating α-linoleic acid in whey protein concentrate by electrospraying. Moreover, these authors reported oil degradation when encapsulating the same formulation by spray drying, and consequently they did not report encapsulation efficiency for this method [34]. According to the results previously reported by Prieto et al., it seems that there is not a significant effect of the heat-stabilization treatment of the whey protein in oil retention, since a similar percentage of oil extraction was observed in the cited previous work [14].

### 3.3. Nitrogen Adsorption and Desorption Isotherms

**Figure 3:**
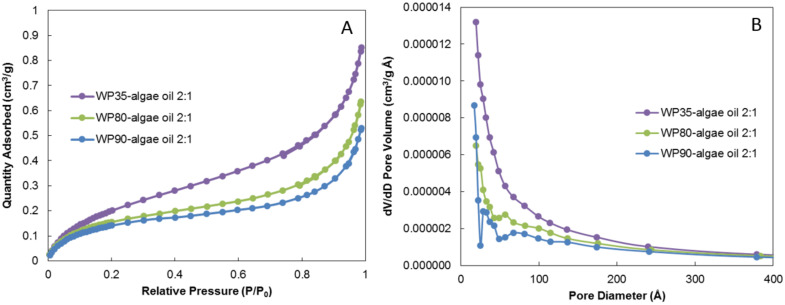
Nitrogen adsorption isotherms (**A**) and pore size distribution (**B**) for algae oil encapsulates in WP35, WP80, and WP90.

The porosity of the particles constitutes an indication of the powder susceptibility to oxidation, since air can enter into contact with the oil through the pores, provoking its oxidation [18]. The porosity, the surface area, and the pore size distribution of the prepared microparticles were analyzed using nitrogen adsorption isotherms, and results are shown in Figure 3 and Table 3. According to the IUPAC classification, the nitrogen adsorption isotherms in Figure 3A correspond to the type II isotherms and type H3 hysteresis loops for the three samples, which means that the microparticles contain slit-shaped pores [35]. From this figure, the amount of adsorbed nitrogen decreased with increasing protein purity. The pore size distribution results are presented in Figure 3B, revealing a similar trend: as the protein purity increased, the pore volume decreased.

The calculated results of surface area, average pore size diameter, and pore volume of the microparticles are gathered in Table 3. The obtained microparticles showed a reduced surface area with increasing protein purity, probably due to the higher number of indentations and to the higher degree of porosity observed for encapsulates prepared with WP35. The average pore diameter results indicated a predominance of the mesopores (pore diameter between 2 and 50 nm) according to the IUPAC classification [35]. The increase in the whey protein purity from WP35 to WP80 did not provoke a significant change in the average pore diameter, whereas it was comparatively reduced for the WP90 encapsulant.

The approximate size of air-constituting molecules is 4 Å [36], so in principle, the pore sizes estimated to be present in the obtained capsules could just allow the passage of air through. This possibility was evaluated through the headspace oxygen depletion test (see Section 3.4). Additionally, these pore sizes could also allow for a certain amount of the oil to leak out through the pores; and the solvent used in the analysis of the extractable oil could extract some oil through the pores, or even provoke the plasticization of the particles, which could be a potential cause for a fraction of the extractable oil observed.

Tavares et al. reported similar average pore diameter, around 5 nm; but higher cumulative volume of pores, around 0.003 cm^3^/g, when producing whey protein isolate and chitosan microparticles encapsulating garlic extract by complex coacervation followed by freeze drying [37]. Larger pore diameters, i.e., 45 nm, were reported by Maia Porte et al. when producing dextrin microparticles by spray drying [38]. The spray-drying technique has also been reported by Amaro et al. to create sugar nanoporous microparticles with much larger specific surface areas, between 40 and 90 m^2^/g [39].

### 3.4. Headspace Oxygen Depletion Test

**Figure 4:**
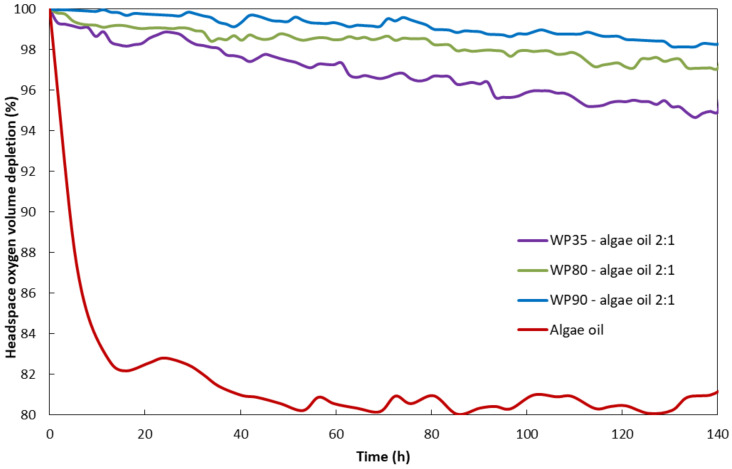
Averaged comparison of the percentage of headspace oxygen volume depletion as a function of time for the neat oil and encapsulates with the three different whey protein contents.

The headspace oxygen depletion of the encapsulates and the neat algae oil were further evaluated by the fluorescence decay methodology at 25 °C and 0% RH for comparative amounts of algae oil to determine the oxygen barrier capacity of the different grades of whey protein used. Figure 4 shows the comparison of the averaged percentage of headspace oxygen volume depletion for the three grades of whey protein and the free algae oil. In the encapsules, the oxygen consumption due to the encapsulating matrix was subtracted, even though this was very small, to consider only the oxygen consumed by the oil. As it can be observed in Figure 4, the algae oil presented the fastest rate of oxygen consumption, which consumed around 18% of the oxygen in approximately 20 h. However, between the different encapsulates, it was observed that the oxygen consumption was the lowest for the sample with the highest whey protein purity. This effect is in agreement with the highest oxygen barrier capacity associated with whey protein, especially in dry conditions [11,40], and to the reduced porosity presented by this sample. In comparison with the heat-stabilized whey protein sample with 80% purity, the herein-produced microparticles present an improved performance, since Prieto et al. reported an oxygen consumption of 7% for the former grade [14].

### 3.5. Accelerated Stability against Oxidation

A comparison of the stability against oxidation provided by the different grades of whey protein used to encapsulate the algae oil with the neat oil was made, exposing the samples to UV light for 10 days. Oil oxidation was assessed in terms of PV and ATR-FTIR spectroscopy.

The formation of primary fatty acid oxidation products, concretely hydroperoxides, was quantified by the PV. The PV of the algae oil at time zero was 1.6 ± 0.9 meq/kg, which is in agreement with the Global Organization for EPA and DHA omega-3s (GOED) [41]. According to the results gathered in Table 2, the observed PV was also dependent on protein purity, being lower as protein purity increased. The very small increase seen in the PV of the sample encapsulated with WP90 is noticeable, which means that the oil did not get oxidized during the room-temperature EAPG process for this sample. A small increase in the peroxide value of the microparticles prepared with WP80 was observed in comparison with the heat-stabilized whey protein 80, reported by Prieto et al. [14], which could be related to the different protective properties of both whey proteins.

**Figure 5:**
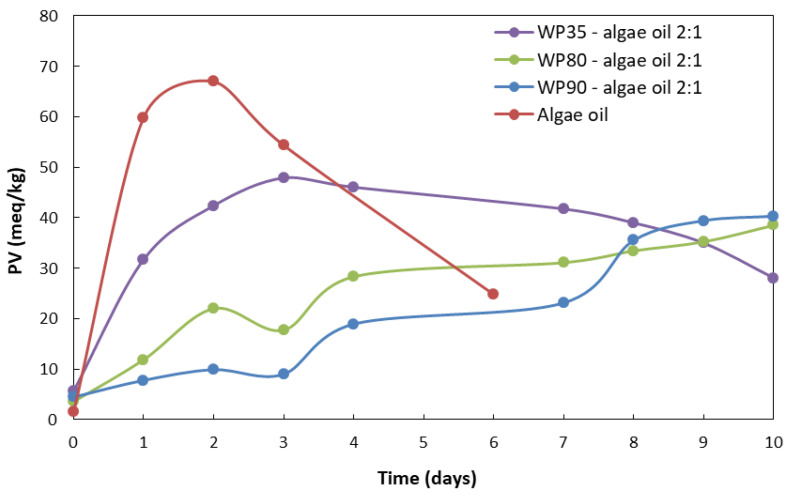
Comparison of the progression of the peroxide value between the encapsulates with the different grades of whey protein and the neat oil during the accelerated oxidation test.

Regarding the results of the evolution of the PV with UV exposure time shown in Figure 5, it could be observed that PV increased rapidly in free algae oil and algae-oil-loaded WP35 microparticles, and then decreased as a result of the chain reactions of the hydroperoxides into secondary oxidation products, such as aldehydes and ketones, responsible for the organoleptic impact [42,43]. The peroxide value of the neat oil could not be measured after the sixth day due to the gelation of the oil. However, in algae-oil-loaded WP80 and WP90 microparticles, the results showed a slow increase in the PV value. This result could be due to the protective properties of whey proteins, which are well-known for their gas barriers and antioxidant capacity [12]. The decomposition of the hydroperoxides that lead to secondary oxidation products was not clearly visible in these two samples.

**Figure 6:**
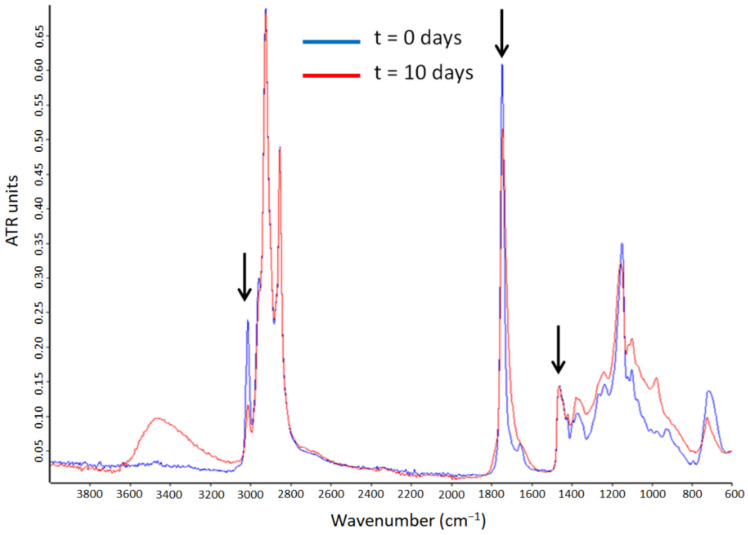
Evaluation of the ATR-FTIR spectra of the algae oil before and after the accelerated oxidation test. Arrows identify the band used as reference, 1456 cm^−1^, and the bands with the most significant changes, 3012 and 1741 cm^−1^.

The peroxide value quantifies the primary oxidation products; however, it does not provide information about the secondary oxidation products responsible of the organoleptic impact [42,43]. In this sense, the generation of secondary oxidation products can be, for instance, ascertained by ATR-FTIR spectroscopy [14]. Thus, the evolution of the spectra of the pure algae oil was analyzed during 10 days of UV light exposure. Figure 6 shows the comparison of the spectra of the pure algae oil during 10 days of UV light exposure. The spectra were normalized to the intensity of the band at 1456 cm^−1^ for comparison purposes [14]. This band was used as an internal standard, since being assigned to the bending vibration of CH_2_ and CH_3_ aliphatic groups, it does not show variations during oxidation. Regarding the main bands in PUFAs, the peak at 3012 cm^−1^ was clearly identified, and it is attributed to the stretching vibrations of alkenes. The relative decrease in the intensity of this band was due to the disappearance of the unsaturations as a result of the oxidation chain reactions [44,45]. The following characteristic band is the one at 1741 cm^−1^, attributed to the stretching of ester and acid groups in triglycerides [44,46]. This band changed its intensity and widened towards lower wavenumbers because of the generation of secondary oxidation products [47]. The bands at ca. 1238 and 1163 cm^−1^, ascribed to the proportion of saturated acyl groups, moved towards higher wavenumbers along the 10 days of UV light exposure, indicating the generation of small saturated acyl molecules [47]. The band at ca. 971 cm^−1^, corresponding to trans-double bonds, increased its intensity, which indicates a rise in the proportion of this kind of bonds during the accelerated oxidation process [46]. Lastly, the band at ca. 705 cm^−1^, assigned to the overlapping of the methylene rocking vibration and the out-of-plane bending vibration of cis-disubstituted olefins, presented an increased intensity during the accelerated oxidation test [47].

**Figure 7:**
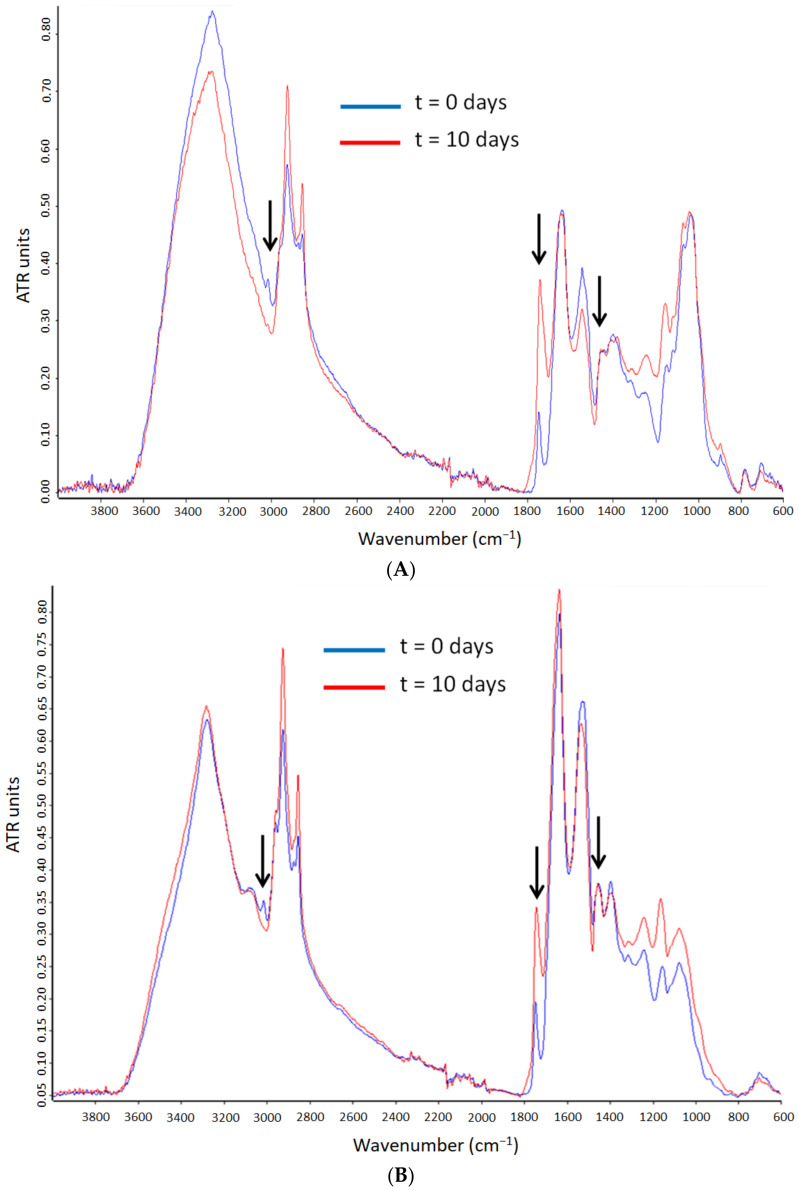

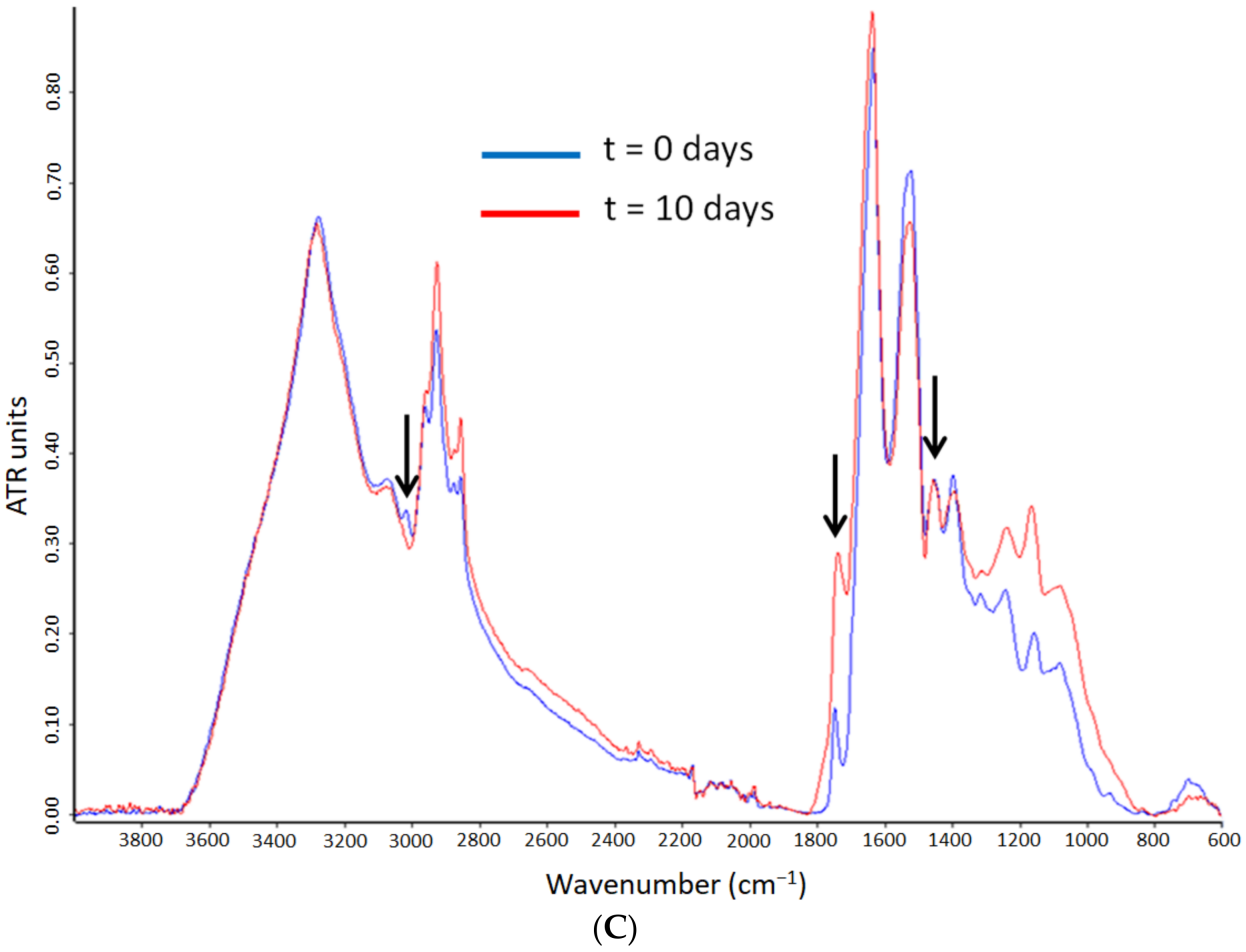
Evolution of the ATR-FTIR spectra of the encapsulates during the accelerated oxidation test. (**A**) WP35-algae oil 2:1; (**B**) WP80-algae oil 2:1; (**C**) WP90-algae oil 2:1. For ease of comparison, the spectra were normalized to the intensity of the band ca. 1456 cm^−1^. Arrows identify the band used as reference, 1456 cm^−1^, and the bands with the most significant changes, 3012 and 1741 cm^−1^.

**Figure 8:**
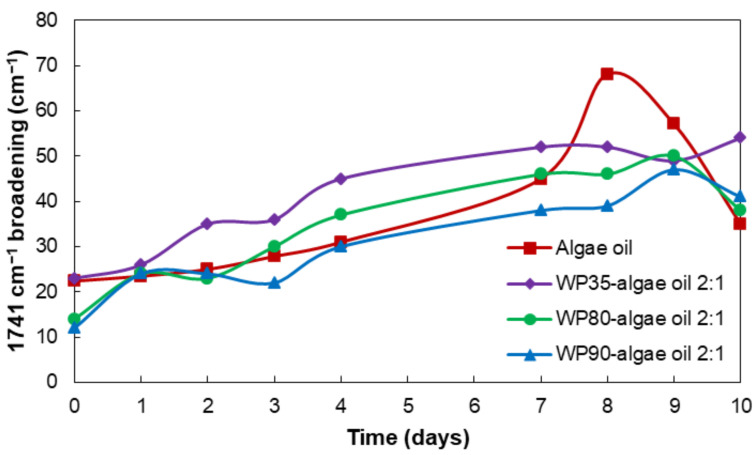
Comparison of the evolution of the 1741 cm^−1^ band broadening for the neat algae oil and the encapsulates, measured at half-height after deconvolution, during the UV accelerated oxidation test.

The ATR-FTIR spectra of the encapsulates were also analyzed along the accelerated oxidation test. Figure 7A–C shows the comparison of the microparticles’ spectra prepared with the different grades of whey protein. The main changes in these spectra were due to the algae oil oxidation. The characteristic bands of proteins at 1650 and 1550 cm^−1^ assigned to the amide I and amide II [48] were clearly visible. The most substantial variations were the reduction in intensity of the band at 3012 cm^−1^ and the band broadening of the 1741 cm^−1^ band. However, this last effect was chosen as the most appropriate to assess the extension of the secondary oxidation reactions, since it does not require normalization to any other spectral feature. Hence, Figure 8 illustrates the 1741 cm^−1^ band broadening at half-maximum intensity during the accelerated oxidation test. The neat algae oil presented a slow increase in the band broadening until the seventh day, after which the broadening rate accelerated and then it decreased rapidly, which could be due to the autocatalytic oxidation chain reactions. The fast increase after the seventh day could be also related to the gelation of the oil, which was also observed after the sixth day in the peroxide value determination. These results appear to confirm the protective ability of WP against UV radiation, resulting in a reduced production of secondary oxidation products [49]. This protective effect was also reported by Prieto et al. in the encapsulation of algae oil into heat-stabilized whey protein 80 [14], and by Escobar-García et al. in the encapsulation of EPA oil into WP80 [15].

**Figure 9:**
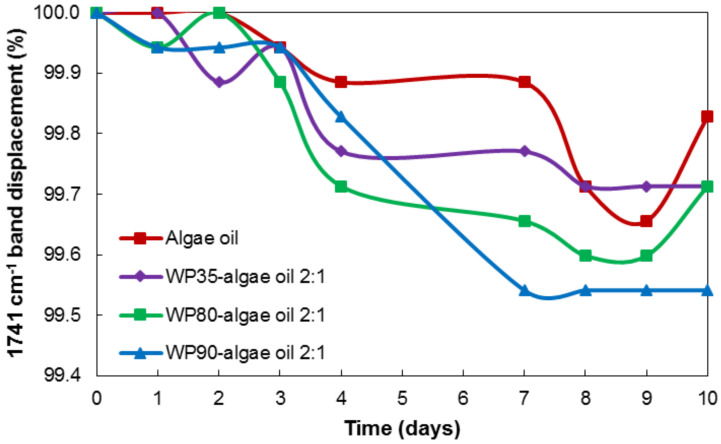
Comparison of the displacement of the 1741 cm^−1^ band for the neat algae oil and the encapsulates during the UV accelerated oxidation test.

Figure 9 shows the displacement of the band of 1741 cm^−1^ during the 10 days of the accelerated oxidation test. During the oxidation, aldehydes and ketones are formed as a consequence of the secondary oxidation reactions. In the case of the encapsulates, the position of the band shifts to lower wavenumbers due to, in our hypothesis, the formation of volatile compounds (i.e., aldehydes and ketones), which may be more pronounced in the case of the neat algae oil. Among the various encapsulates, the WP90 shows the highest decrease in the position of the band, suggesting a lower extent for the secondary oxidation processes.

### 3.6. Thermogravimetric Analysis

**Figure 10:**
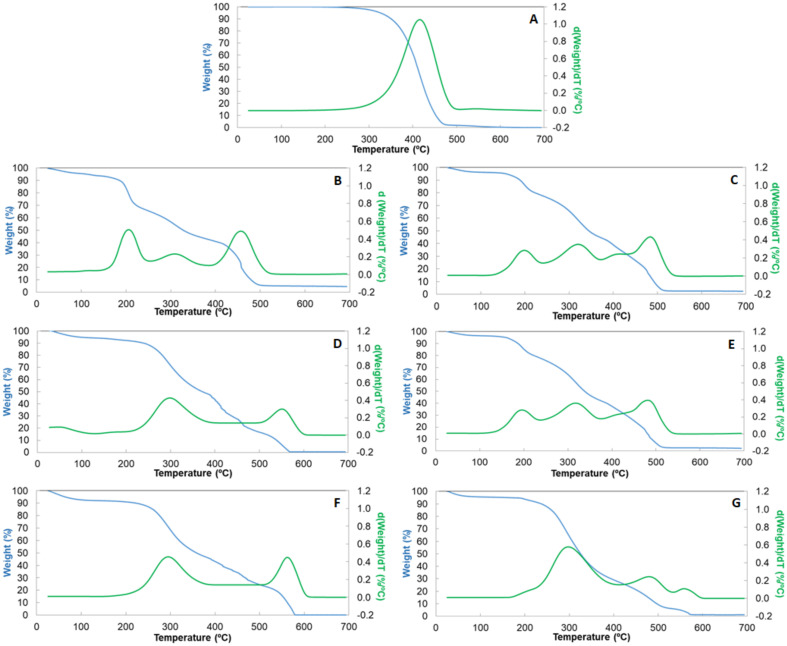
TGA curves in the presence of oxygen for (**A**) algae oil, (**B**) WP35, (**C**) WP35-loaded algae oil, (**D**) WP80, (**E**) WP80-loaded algae oil, (**F**) WP90, (**G**) WP90-loaded algae oil.

Thermogravimetric analysis was used to compare the thermal stability of the free algae oil, the pure encapsulating materials, and the encapsulated oil with different whey protein purities. The analysis was performed in air. Figure 10 presents the obtained results. Algae oil was stable until 215 °C, obtaining a total weight loss between 215 and 600 °C, which could be due to the loss of volatile compounds until 350 °C and pyrolysis phenomena at higher temperatures [50], as shown in Figure 10A.

Regarding the encapsulants (Figure 10B,D,F), all of them showed the first thermal event below 100 °C, which is attributed to humidity. The second thermal event around 250–275 °C was due to breakage of the covalent peptide bond in the amino acid. In the case of WP35, it occurred at a lower temperature, probably due to the high content of lactose, which shows a significant weight loss between 150–160 °C due to the loss of crystal water [51]. The third thermal event took place at above 340 °C due to the cleavage of S-S, O-N, and O-O linkages from protein molecules, as a consequence of the decomposition of proteins [52]. The last thermal event around 450–500 °C was due to pyrolysis of the sample. These results are in agreement with Abbastabr et al., who observed a similar degradation profile for WPI microparticles prepared by spray drying [53].

The thermal stability of the materials was calculated as the temperature at which about 5% (T_5%_) of mass loss occurs after the moisture loss. In this sense, WP35 showed reduced thermal stability, showing the T_5%_ at 155 °C, whereas for WP80 and WP90 this temperature was around 190 °C. Regarding the encapsulates, the addition of the algae oil decreased the thermal stability compared to the pure protein. Hence, oil-loaded microparticles made of WP35 and 80 showed a similar thermal stability, with T_5%_ being around 157 °C, whereas for WP90 it was 170 °C. This behavior could be due to the combined effects of differences in protein purity, but also due to microparticles’ internal structure when containing algae oil (as shown in Figure 2), which was similar for WP35 and WP80, but different for WP90.

## 4. Conclusions

In this work, the effect of whey protein purity on algae oil protection via room-temperature EAPG encapsulation was studied for the first time. The protein purity seemed to have an effect on the sphericity and roughness of the microparticles, thermal stability, as well as in the porosity, which affected the stability against oxidation. Peroxide values confirmed the superior protective effect of the protein against oxidation, being consistent with the porosity and the oxygen permeation results. Additionally, when the protein content was increased, enhanced thermal stability was observed for the encapsulates. However, the content of lactose favored the oil retention, but affected the oxidative stability of the oil inside the microparticle, probably due to the increased porosity and a lower oxygen barrier capacity. The obtained results demonstrate the importance of the selection of adequate wall material together with the encapsulation method. Thus, whey proteins with protein content higher than or equal to 80% are recommended for optimal oil stability.

## Figures and Tables

**Table 1:** Composition of the grades of whey protein used as wall materials.

	WP35	WP80	WP90
Protein (%)	35	80	90
Ash (%)	≤7.5	≤5	≤3.0
Fat (%)	≤3.5	≤10	≤1.5
Lactose (%)	≥50	≤10	≤2.0
Moisture (%)	≤5	≤6	≤5.0

**Table 2:** Characteristics of the emulsions and particles encapsulating algae oil within the different grades of whey protein through the room-temperature EAPG method. EO means extractable oil; PV means peroxide value.

	Emulsion Mean Droplet Size (µm)	Average Particle Size (µm)	EO (%)	PV (meq/kg)
WP35-algae oil 2:1	0.530 ± 0.071	3.07 ± 1.65	17 ± 2	6.7 ± 0.6
WP80-algae oil 2:1	0.661 ± 0.001	3.68 ± 1.71	35 ± 4	3.6 ± 0.3
WP90-algae oil 2:1	0.636 ± 0.001	3.09 ± 2.13	35 ± 2	1.9 ± 0.2

**Table 3:** Surface area and porosity of algae oil-loaded microparticles prepared with different protein purity in whey protein by EAPG.

Sample	BET Surface Area (m^2^/g)	Average Pore Diameter by BET (nm)	BJH Adsorption Cumulative Surface Area of Pores (m^2^/g)	BJH Desorption Cumulative Surface Area of Pores (m^2^/g)	BJH Adsorption Cumulative Volume of Pores (cm^3^/g)	BJH Desorption Cumulative Volume of Pores (cm^3^/g)
WP35-algae oil 2:1	0.85	6.07	0.56	0.76	0.0011	0.0013
WP80-algae oil 2:1	0.64	6.06	0.32	0.59	0.0008	0.0010
WP90-algae oil 2:1	0.57	5.63	0.28	0.45	0.0007	0.0008
